# Supplementary material for: Scanning Probe Spectroscopy of WS2/Graphene Van Der Waals Heterostructures
Source: Nanomaterials (Basel). 2020 Dec 11;10(12):2494. doi: 10.3390/nano10122494 (PMC7762982; doi:10.3390/nano10122494)
Supplement: Supplementary file 1 [file nanomaterials-10-02494-s001.pdf]

nanomaterials

MDPI

Article

Scanning Probe Spectroscopy of WS_2/Graphene Van Der Waals Heterostructures

Franco Dinelli [1,*], Filippo Fabbri [2,3,4], Stiven Forti [4], Camilla Coletti [4,5], Oleg V. Kolosov [6] and Pasqualantonio Pingue [2,3]

1. CNR, Istituto Nazionale di Ottica, via Moruzzi 1, 56124 Pisa, Italy
2. NEST, Scuola Normale Superiore, Piazza San Silvestro 12, 56127 Pisa, Italy; filippo.fabbri@nano.cnr.it (F.F.); pasqualantonio.pingue@sns.it (P.P.)
3. CNR, NEST, Istituto di Nanoscienze, Piazza San Silvestro 12, 56127 Pisa, Italy
4. CNI@NEST, Istituto Italiano di Tecnologia, Piazza San Silvestro 12, 56127 Pisa, Italy; stiven.forti@iit.it (S.F.); Camilla.Coletti@iit.it (C.C.)
5. Graphene Labs, Istituto Italiano di Tecnologia, Via Morego 30, I-16163 Genova, Italy
6. Department of Physics, University of Lancaster, Bailrigg, Lancaster LA1 4YB, UK; o.kolosov@lancaster.ac.uk

* Correspondence: franco.dinelli@ino.cnr.it; Tel.: +39-050 315 2564

Received: 10 November 2020; Accepted: 8 December 2020; Published: 11 December 2020

Abstract: In this paper, we present a study of tungsten disulfide (WS_2) two-dimensional (2D) crystals, grown on epitaxial Graphene. In particular, we have employed scanning electron microscopy (SEM) and μRaman spectroscopy combined with multifunctional scanning probe microscopy (SPM), operating in peak force–quantitative nano mechanical (PF-QNM), ultrasonic force microscopy (UFM) and electrostatic force microscopy (EFM) modes. This comparative approach provides a wealth of useful complementary information and allows one to cross-analyze on the nanoscale the morphological, mechanical, and electrostatic properties of the 2D heterostructures analyzed. Herein, we show that PF-QNM can accurately map surface properties, such as morphology and adhesion, and that UFM is exceptionally sensitive to a broader range of elastic properties, helping to uncover subsurface features located at the buried interfaces. All these data can be correlated with the local electrostatic properties obtained via EFM mapping of the surface potential, through the cantilever response at the first harmonic, and the dielectric permittivity, through the cantilever response at the second harmonic. In conclusion, we show that combining multi-parametric SPM with SEM and μRaman spectroscopy helps to identify single features of the WS_2/Graphene/SiC heterostructures analyzed, demonstrating that this is a powerful tool-set for the investigation of 2D materials stacks, a building block for new advanced nano-devices.

Keywords: 2DM; tungsten disulfide; epitaxial graphene; SiC; PF-QNM; UFM; EFM

In Figure S1, we first present the topography of a fresh sample taken within few hours from the exposure to air. The characteristics steps indicated in Figure 3 are also shown here. The height of these steps is equal to 0.5 ± 0.2 nm (point 1), 0.6 ± 0.2 nm (point 3), 0.8 ± 0.2 nm (points 2 and 2′). The height of the bumps ranges from 10 to 30 nm.

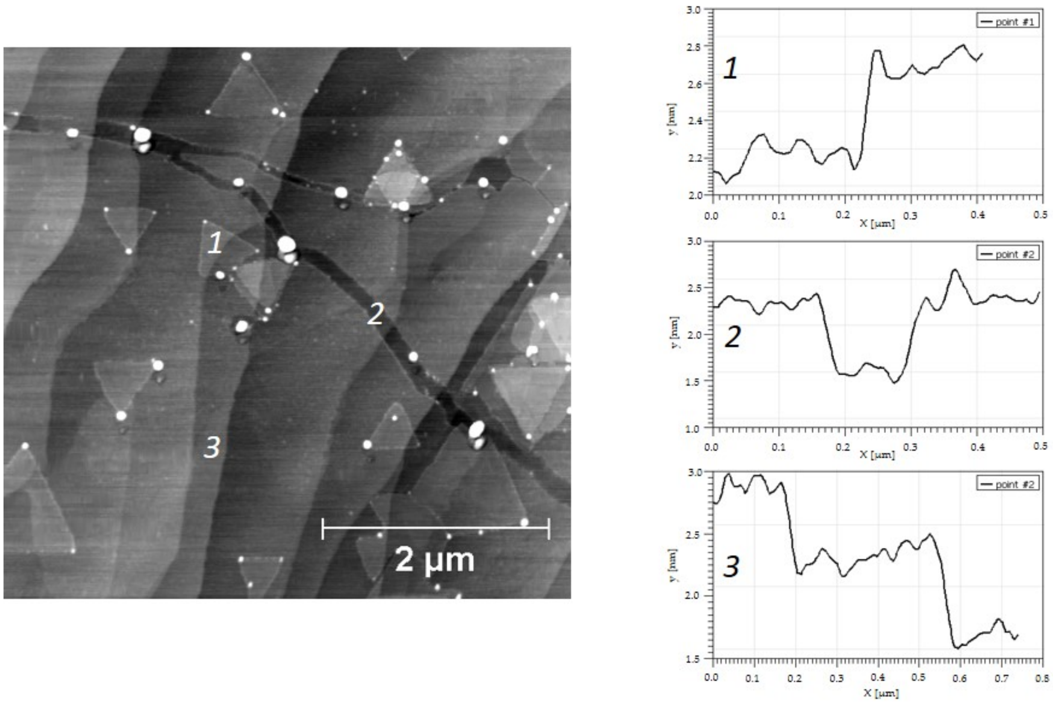

Figure S1. Topographical data obtained on a fresh sample, grown in the same conditions as the one of Figure 3

In Figure S2, we present some data obtained operating in contact mode on the same sample reported in Figure 4. Fresh samples are rather stable under contact and no wear is visible under certain force values. After 2 days, some wear occurs at the oxidized borders and also in other areas where loose material is probably present. This indicates that the oxidized regions are weaker and less stable. After 120 days, wear can occur almost everywhere. This indicates that the oxidation is widespread and has weakened the whole layer, also the interfaces with epiGr.

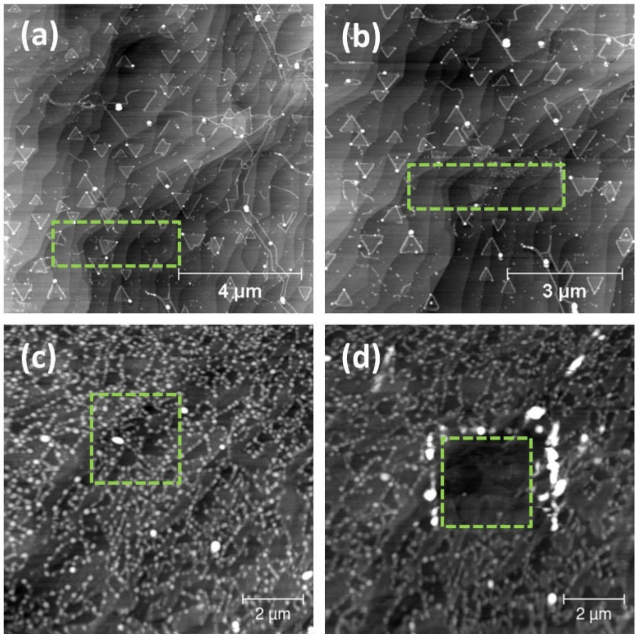

Figure S2. Morphology data of a sample after 2 (**a**,**b**) and 120 (**c**,**d**) days. The images (**a**,**c**) were taken before while (**b**,**d**) after scanning smaller areas in contact mode with no ultrasonic excitation. Wear of the oxidized regions clearly occurs.

In order to optimize the KPFM contrast, we have varied the applied AC bias. In our experiments, we have not noticed any clear change upon increasing the voltage from 0.1 up to 2 V, except for the absolute value of the KPFM response. However, if the applied bias exceeds a certain threshold value, the local morphology may dramatically change.

In Figure S3, we present the topography of a sample area before and after a scanning in KPFM mode at 5 V: along the borders of the WS_2 crystals, some additional bumps have appeared. They are probably due to local anodic oxidation process occurring between tip and specimen [1S], accelerating the standard oxidation kinetic. It is well known, that in air a water bridge forms between tip and surface, whose lateral dimensions depend on the relative humidity value of the environment. The threshold value for the occurrence of oxidation may depend on parameters such as the tip material employed, the scanning parameters and the relative humidity itself [2S]. This behaviour additionally confirms the oxide nature of the decoration of the WS_2 crystal edges.

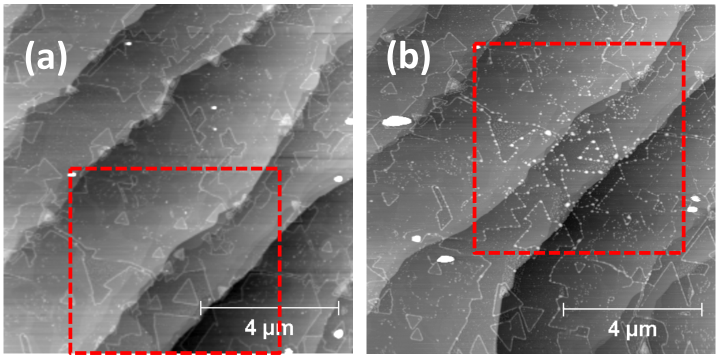

Figure S3. Morphology data obtained (**a**) before and (**b**) after operating in KPFM while applying to the tip an AC bias voltage of 5 V in the highlighted region.

In Figure S4, we finally present the topography of an oxidized WS_2 crystal before and after scanning in contact mode to scratch away the oxidized border. After the removal, we have measured the thickness of the area and have found that it is almost null. This indicate the complete dissolution of the WS_2 terrace.

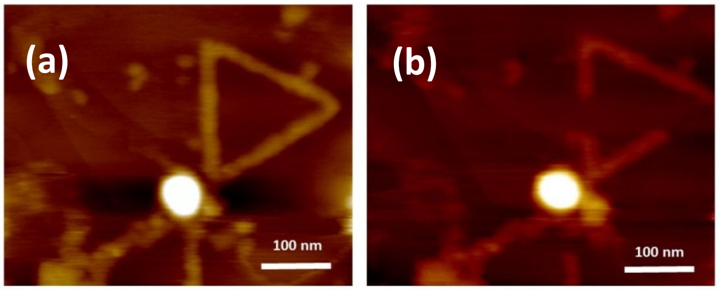

Figure S4. An oxidized WS_2 island before (**a**) and after (**b**) scratching its edges with the SPM tip: no residual WS_2 layer can be observed after removing the edge.

References

S1. Dagata, J.A.; Schneir, J.; Harary, H.H.; Evans, C.J.; Postek, M.T.; Bennett, J. Modification of hydrogen-passivated silicon by a scanning tunneling microscope operating in air. *Appl. Phys. Lett.* **1990**, *56*, 2001

S2. Avouris, P.; Hertel, T.; Martel, R. Atomic force microscope tip-induced local oxidation of silicon: kinetics, mechanism, and nanofabrication. *Appl Phys Lett.* **1997**, *71*, 285.
